# Association between lipid accumulation product index and the prevalence of cardiovascular diseases induced by 2-hydroxyfluorene: A cross-sectional study from the NHANES (2005–2016)

**DOI:** 10.1097/MD.0000000000041681

**Published:** 2025-03-14

**Authors:** Zhuofan Deng, Xinling Tian, Tian Xiong, Qingsong Mao, Xiaoyi Zhu, Yuzhe Kong

**Affiliations:** a Department of Hepatobiliary Surgery, The Chengdu Sixth People’s Hospital, Chengdu, China; b Xiangya School of Medicine, Central South University, Changsha, China; c Hepatobiliary Pancreatic Surgery, Banan Hospital Affiliated to Chongqing Medical University, Chongqing, China.

**Keywords:** angina pectoris, congestive heart failure, coronary heart diseases, lipid accumulation product, myocardium infarction, polycyclic aromatic hydrocarbon, stroke

## Abstract

This research explored the effects of 2-hydroxyfluorene exposure on the incidence of cardiovascular diseases (CVDs), particularly examining the mediating role of the lipid accumulation product (LAP) index. Utilizing data from the National Health and Nutrition Examination Survey spanning 2005 to 2018, this analysis evaluated the impact of 2-hydroxyfluorene on CVDs prevalence employing a variety of statistical methods. Logistic regression was applied to investigate relationships within polycyclic aromatic hydrocarbon mixtures, complemented by Bayesian Kernel Machine Regression. Additionally, a mediation analysis explored the influence of the LAP index in moderating the effects of 2-hydroxyfluorene on CVDs prevalence. The research also detailed the link between 2-hydroxyfluorene exposure and specific CVDs such as congestive heart failure, myocardial infarction, angina pectoris, and coronary heart disease, including their mediated impacts. Involving 3653 participants, the study detected a robust positive correlation between 2-hydroxyfluorene exposure and overall CVD risk (OR [95%CI] = 393.5173 [23.6978–6534.5979], *P* < .0001). This association extended to the prevalence of specific types of CVDs. The LAP index served as a mediator in the connection between 2-hydroxyfluorene exposure and the prevalence of total CVD, congestive heart failure, myocardial infarction, coronary heart disease, and stroke, with mediation percentages of 12%, 10%, 100%, 5%, and 9%, respectively. These results underscore a significant link between 2-hydroxyfluorene exposure and increased prevalence of CVDs, with the LAP index playing a crucial mediating role.

## 1. Introduction

Cardiovascular diseases (CVD) are leading causes of death and decreased quality of life globally, resulting in significant health care costs and reduced wellness.^[1,2]^ Identifying early predictors of CVD risk is essential for implementing preventive strategies.

Polycyclic aromatic hydrocarbons (PAHs), produced by human activities like incomplete combustion of fossil fuels and natural events such as wildfires and volcanic eruptions, are common environmental contaminants.^[3,4]^ These pollutants bind to airborne particulate matter (PM), including PM10 and PM2.5. Previous studies have connected exposure to PM with hypertension^[5,6]^ and highlighted PAHs’ efficient absorption through skin, ingestion, and inhalation due to their lipid solubility.^[7,8]^

Long-term exposure to PAHs is linked to increased risks of oxidative stress (OS), metabolic syndrome (MS), chronic obstructive pulmonary disease, and various cancers.^[9,10]^ Evidence suggests that PAH exposure raises blood pressure, thereby significantly influencing CVD risk.^[11,12]^ The ensuing OS, together with vasoconstriction and dysfunction of endothelial cells, is thought to contribute to PAH-related cardiovascular impacts.^[13]^

Introduced by Kahn in 2005, the lipid accumulation product (LAP) index has been recognized as an accurate measure of lipid accumulation and visceral adiposity, surpassing traditional body fat metrics.^[14]^ Subsequent research has positioned LAP as a potent predictor of CVD, MS, and diabetes.^[15–19]^

Given these dynamics, it is conceivable that PAH exposure could affect CVD incidence through the LAP index. This study employs National Health and Nutrition Examination Survey (NHANES) data to examine the mediated effects of LAP indicators, supporting the proposed associations.

## 2. Methods

### 2.1. Study population

The research utilized the NHANES data, overseen by the CDC’s National Center for Health Statistics. This survey assesses the health and nutrition of the noninstitutionalized U.S. populace, employing a stratified multistage sampling approach to accurately represent the demographic makeup of the country (https://www.cdc.gov/nchs/nhanes/index.htm). The collection of initial health and nutritional information was conducted via direct interviews, assessments in mobile examination centers, and a variety of laboratory tests.

### 2.2. Ethical approval

Informed consent was obtained from all participants. Ethic approval received from NCHS Ethics Review Board in accordance with the Declaration of Helsinki (Protocol #2011-17 and Protocol #2005-06).

### 2.3. Inclusion and exclusion criteria

**Figure 1.**
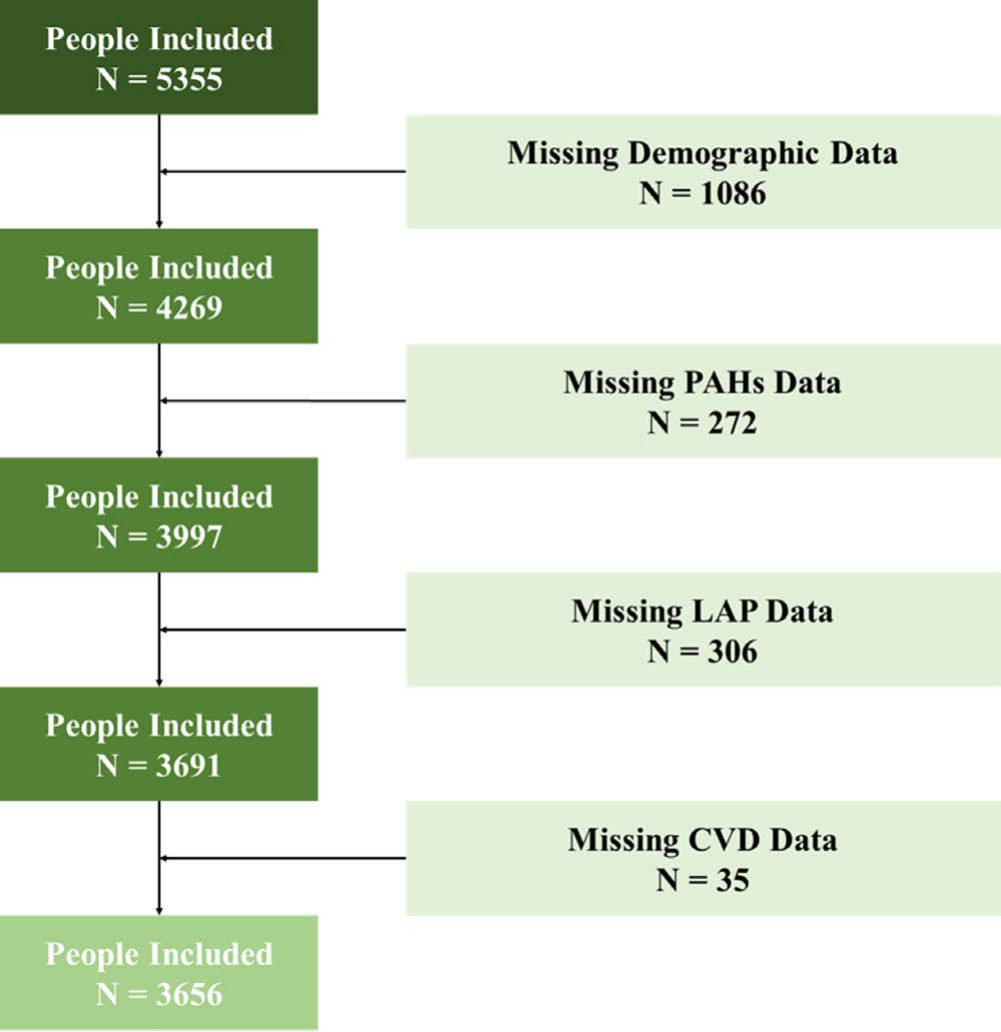
Study flowchart.

Initially, 5355 participants were included, where 1699 participants were excluded because of missing data. Thus, 3656 participants were finally included (Fig. 1).

Specifically, 1086 participants missed demographic data. After excluding them, 272 participants missed PAHs data. Then, we excluded 306 participants because of the lack of LAP data. After that, 35 lacked CVD diagnosis data. Finally, we included 3656 participants.

### 2.4. Exposure measurement

This method measures various OH-PAH analytes, including monohydroxylated compounds such as 1-hydroxynaphthalene, 2-hydroxynaphthalene, 2-hydroxyfluorene, 3-hydroxyfluorene, 1-hydroxyphenanthrene, and 2- and 3-hydroxyphenanthrene, as well as 1-hydroxypyrene. The process begins with enzymatic hydrolysis to release conjugated OH-PAH metabolites in urine, followed by on-line solid phase extraction. The compounds are then detected and quantified using isotope dilution high-performance liquid chromatography paired with tandem mass spectrometry (on-line SPE-HPLC-MS/MS).

### 2.5. Outcome measurement

This method measures various OH-PAH analytes, including monohydroxylated compounds such as 1-hydroxynaphthalene, 2-hydroxynaphthalene, 2-hydroxyfluorene, 3-hydroxyfluorene, 1-hydroxyphenanthrene, and 2- and 3-hydroxyphenanthrene, as well as 1-hydroxypyrene. The process begins with enzymatic hydrolysis to release conjugated OH-PAH metabolites in urine, followed by on-line solid phase extraction. The compounds are then detected and quantified using isotope dilution high-performance liquid chromatography paired with tandem mass spectrometry (on-line SPE-HPLC-MS/MS).

### 2.6. Covariates

Considered our analysis included essential covariates recognized in previous studies,^[20,21]^ such as age, gender, race/ethnicity, educational attainment, marital status, family poverty income ratio (PIR), alcohol intake, smoking habits, diabetes, and hypertension.

Race/ethnicity in NHANES is divided into Mexican American, other Hispanic, non-Hispanic white, non-Hispanic black, and a group including non-Hispanic Asians and multiracial individuals. Educational levels span from less than ninth grade to a college degree or higher. Marital status ranges from married to undisclosed, reflecting diverse family structures. PIR evaluates annual income relative to poverty thresholds adjusted by family size.

Alcohol consumption is determined by whether individuals drink at least 12 alcoholic beverages yearly. Smoking status is defined by smoking over 100 cigarettes in one’s lifetime. Both diabetes and hypertension are self-reported and confirmed by health professionals.

### 2.7. Statistical analysis

Our statistical methods employed the Kruskal–Wallis test for continuous data and Fisher exact test for sparse categorical data, with PAHs values log-transformed for normalization.

Descriptive analyses initially explored the relationship between individual PAHs and CVD. Further examinations of PAH mixtures involved logistic regression and Bayesian Kernel Machine Regression (BKMR) to assess nonlinear interactions. Nonparametric bootstrapping (n = 1000) was used for mediation analysis to pinpoint direct and indirect effects.

All statistical procedures accounted for demographic variables^[22,23]^ and were executed in R software version 4.3.3, aiming for statistical significance at *P* < .05.

## 3. Results

### 3.1. General information

The research involved 3656 individuals, segmented into 2 groups based on CVD prevalence, with a diagnosed rate of 9.66% (Table 1). Notable differences (*P* < .05) were observed across gender, age, race/ethnicity, education, marital status, family PIR, hypertension, diabetes, smoking status, 2-hydroxyfluorene, 1-hydroxynaphthalene, and LAP index between the groups.

**Table 1:** General information.

	Non-CVDs	CVD	*P*
*Population*	3303	353	
*Gender*			**.0000**
Male	1629 (49.32%)	218 (61.76%)	
Female	1674 (50.68%)	135 (38.24%)	
*Age*	47.04 ± 17.2	65.1 ± 13.01	**.0000**
*Race and ethnicity*			**.0000**
Mexican American	532 (16.11%)	34 (9.63%)	
Other Hispanic	326 (9.87%)	33 (9.35%)	
Non-Hispanic white	1484 (44.93%)	210 (59.49%)	
Non-Hispanic black	630 (19.07%)	62 (17.56%)	
Other race-including multi-racial	331 (10.02%)	14 (3.97%)	
*Educational background*			**.0000**
Less than 9th grade	285 (8.63%)	60 (17%)	
9–11th grade (includes 12th grade with no diploma)	456 (13.81%)	59 (16.71%)	
High school graduate/GED or equivalent	763 (23.1%)	82 (23.23%)	
Some college or AA degree	959 (29.03%)	82 (23.23%)	
College graduate or above	840 (25.43%)	70 (19.83%)	
*Marital status*			**.0000**
Married	1731 (52.41%)	203 (57.51%)	
Widowed	203 (6.15%)	52 (14.73%)	
Divorced	348 (10.54%)	52 (14.73%)	
Separated	103 (3.12%)	9 (2.55%)	
Never married	645 (19.53%)	21 (5.95%)	
Living with partner	273 (8.27%)	16 (4.53%)	
*PIR*	2.61 ± 1.63	2.27 ± 1.53	**.0000**
*Drinking*			.6886
No	879 (26.61%)	98 (27.76%)	
Yes	2424 (73.39%)	255 (72.24%)	
*Hypertension*			**.0000**
No	2235 (67.67%)	100 (28.33%)	
Yes	1068 (32.33%)	253 (71.67%)	
*Diabetes*			**.0000**
No	2980 (90.22%)	236 (66.86%)	
Yes	323 (9.78%)	117 (33.14%)	
*Smoking*			**.0000**
No	1843 (55.8%)	138 (39.09%)	
Yes	1460 (44.2%)	215 (60.91%)	
*PAHs*			
1-Hydroxynaphthalene	0.29 ± 0.12	0.32 ± 0.12	**.0003**
2-Hydroxynaphthalene	0.49 ± 0.16	0.5 ± 0.16	.3817
3-Hydroxyfluorene	0.43 ± 0.17	0.45 ± 0.18	.0645
2-Hydroxyfluorene	0.42 ± 0.16	0.44 ± 0.17	**.0092**
1-Hydroxyphenanthrene	0.4 ± 0.12	0.41 ± 0.12	.7515
1-Hydroxypyrene	0.43 ± 0.13	0.42 ± 0.13	.0999
*LAP*	0.53 ± 0.1	0.57 ± 0.09	**.0001**

Bold values indicate *P* < .05.

CVD = cardiovascular disease, PIR = poverty income ratio.

### 3.2. Association between 2-hydroxyfluorene in PAHs mixtures and the prevalence of CVDs and each specific CVD assessed by logistic model

The study utilized logistic regression to examine the impact of 2-hydroxyfluorene within a PAH mixture on CVD prevalence. The findings showed a significant positive link (Table 2) with overall CVD prevalence (OR [95%CI] = 393.5173 [23.6978–6534.5979], *P* < .0001), and individual CVDs like congestive heart failure (OR [95%CI] = 464.8142 [2.9371–73,559.3865], *P* = .0175), myocardial infarction (OR [95%CI] = 420.8088 [7.6351–23,192.8852], *P* = .0031), angina pectoris (OR [95%CI] = 906.5597 [5.6975–144,246.7515], *P* = .0085), coronary heart disease (OR [95%CI] = 1795.8476 [24.2944–132,749.1956], *P* = .0006), and stroke (OR [95%CI] = 734.4762 [7.4747–72,171.0235], *P* = .0048). This association persisted even after adjusting for covariates (Table 3).

**Table 2:** Association between 2-hydroxyfluorene in PAHs mixtures and the prevalence of CVDs and each specific CVD assessed by logistic model (unadjusted).

Variables	Single factor					Multiple factor				
	β	S.E	Z	*P*	OR (95%CI)	β	S.E	Z	*P*	OR (95%CI)
*Cardiovascular diseases*										
1-Hydroxynaphthalene	1.6727	0.447	3.7423	**.0002**	5.3268 (2.2182–12.7919)	1.8319	0.5953	3.0775	**.0021**	6.2459 (1.9449–20.0576)
2-Hydroxynaphthalene	0.3126	0.3526	0.8867	.3753	1.3670 (0.6850–2.7281)	-0.525	0.5216	‐1.0066	.3141	0.5915 (0.2128–1.6442)
3-Hydroxyfluorene	0.6116	0.3183	1.9211	.0547	1.8433 (0.9877–3.4400)	-2.8139	1.208	-2.3294	**.0198**	0.0600 (0.0056–0.6400)
2-Hydroxyfluorene	0.9481	0.3478	2.726	**.0064**	2.5808 (1.3053–5.1029)	5.9751	1.4336	4.168	**<.0001**	393.5173 (23.6978–6534.5979)
1-Hydroxyphenanthrene	0.1409	0.4533	0.3108	.756	1.1513 (0.4735–2.7991)	-0.8204	0.8051	-1.019	.3082	0.4402 (0.0909–2.1330)
1-Hydroxypyrene	-0.7855	0.4499	-1.7458	.0809	0.4559 (0.1888–1.1012)	-3.4555	0.7902	-4.3731	**<.0001**	0.0316 (0.0067–0.1486)
*Congestive heart failure*										
1-Hydroxynaphthalene	2.1365	0.7701	2.7741	**.0055**	8.4696 (1.8720–38.3188)	1.6068	1.0378	1.5483	.1216	4.9866 (0.6523–38.1195)
2-Hydroxynaphthalene	1.3833	0.6452	2.1439	**.032**	3.9879 (1.1260–14.1240)	0.9342	0.9415	0.9922	.3211	2.5451 (0.4021–16.1097)
3-Hydroxyfluorene	1.0723	0.5671	1.891	.0586	2.9222 (0.9616–8.8798)	-2.6005	2.1607	-1.2036	.2288	0.0742 (0.0011–5.1260)
2-Hydroxyfluorene	1.3972	0.6223	2.2452	**.0248**	4.0437 (1.1942–13.6922)	6.1416	2.5838	2.377	**.0175**	464.8142 (2.9371–73,559.3865)
1-Hydroxyphenanthrene	-0.0677	0.8262	-0.0819	.9347	0.9345 (0.1851–4.7187)	-2.471	1.4873	-1.6614	.0966	0.0845 (0.0046–1.5590)
1-Hydroxypyrene	-0.3863	0.813	-0.4751	.6347	0.6796 (0.1381–3.3438)	-3.3332	1.4329	-2.3262	**.02**	0.0357 (0.0022–0.5917)
*Myocardium infarction*										
1-Hydroxynaphthalene	0.5136	0.6771	0.7584	.4482	1.6712 (0.4433–6.3009)	1.5472	0.8544	1.8108	.0702	4.6982 (0.8803–25.0739)
2-Hydroxynaphthalene	-1.3234	0.5139	-2.575	**.01**	0.2662 (0.0972–0.7290)	-1.8476	0.7562	-2.4432	**.0146**	0.1576 (0.0358–0.6939)
3-Hydroxyfluorene	-0.3379	0.4798	-0.7044	.4812	0.7132 (0.2785–1.8265)	-2.5828	1.7629	-1.4651	.1429	0.0756 (0.0024–2.3926)
2-Hydroxyfluorene	0.0269	0.5161	0.0521	.9584	1.0273 (0.3735–2.8250)	6.0422	2.0457	2.9537	**.0031**	420.8088 (7.6351–23,192.8852)
1-Hydroxyphenanthrene	-0.0781	0.6595	-0.1185	.9057	0.9248 (0.2539–3.3684)	1.8433	1.1301	1.6311	.1029	6.3173 (0.6896–57.8710)
1-Hydroxypyrene	-2.8253	0.6812	-4.1476	**<.0001**	0.0593 (0.0156–0.2253)	-6.3001	1.0952	-5.7527	**<.0001**	0.0018 (0.0002–0.0157)
*Angina pectoris*										
1-Hydroxynaphthalene	1.4881	0.8133	1.8298	.0673	4.4289 (0.8995–21.8055)	1.9339	1.0459	1.8489	.0645	6.9162 (0.8904–53.7237)
2-Hydroxynaphthalene	-0.2547	0.6507	-0.3913	.6956	0.7752 (0.2165–2.7753)	-1.9092	0.9824	-1.9433	.052	0.1482 (0.0216–1.0165)
3-Hydroxyfluorene	0.6122	0.5852	1.0461	.2955	1.8445 (0.5858–5.8080)	-3.2373	2.2025	-1.4698	.1416	0.0393 (0.0005–2.9433)
2-Hydroxyfluorene	0.9682	0.6382	1.517	.1293	2.6332 (0.7538–9.1990)	6.8097	2.5866	2.6327	**.0085**	906.5597 (5.6975–144,246.7515)
1-Hydroxyphenanthrene	0.1577	0.8369	0.1884	.8505	1.1708 (0.2270–6.0378)	-1.5726	1.4672	-1.0718	.2838	0.2075 (0.0117–3.6806)
1-Hydroxypyrene	-0.3806	0.8251	-0.4613	.6446	0.6834 (0.1356–3.4439)	-1.8266	1.4364	-1.2716	.2035	0.1610 (0.0096–2.6879)
*Coronary heart disease*										
1-Hydroxynaphthalene	1.7568	0.6816	2.5774	**.01**	5.7939 (1.5233–22.0374)	1.665	0.9359	1.7791	.0752	5.2857 (0.8443–33.0920)
2-Hydroxynaphthalene	-0.0375	0.5518	-0.068	.9458	0.9632 (0.3266–2.8405)	-2.1027	0.8491	-2.4763	**.0133**	0.1221 (0.0231–0.6450)
3-Hydroxyfluorene	1.1353	0.4877	2.3276	**.0199**	3.1121 (1.1964–8.0950)	-2.4533	1.8872	-1.2999	.1936	0.0860 (0.0021–3.4752)
2-Hydroxyfluorene	1.5531	0.5347	2.9048	**.0037**	4.7262 (1.6573–13.4781)	7.4932	2.1954	3.4131	**.0006**	1795.8476 (24.2944–132,749.1956)
1-Hydroxyphenanthrene	0.6726	0.7064	0.9522	.341	1.9594 (0.4907–7.8237)	-1.0213	1.2416	-0.8225	.4108	0.3601 (0.0316–4.1050)
1-Hydroxypyrene	-0.3646	0.6993	-0.5213	.6021	0.6945 (0.1764–2.7348)	-3.3992	1.2041	-2.823	**.0048**	0.0334 (0.0032–0.3537)
*Stroke*										
1-Hydroxynaphthalene	2.7073	0.6738	4.0182	**<.0001**	14.9890 (4.0018–56.1419)	2.4753	0.869	2.8485	**.0044**	11.8859 (2.1644–65.2712)
2-Hydroxynaphthalene	1.4655	0.5814	2.5205	**.0117**	4.3298 (1.3853–13.5326)	0.92	0.8546	1.0765	.2817	2.5093 (0.4700–13.3973)
3-Hydroxyfluorene	1.1307	0.51	2.2173	**.0266**	3.0979 (1.1402–8.4167)	-2.6622	1.9453	-1.3685	.1712	0.0698 (0.0015–3.1601)
2-Hydroxyfluorene	1.4516	0.5601	2.5919	**.0095**	4.2701 (1.4247–12.7985)	6.5992	2.3407	2.8193	**.0048**	734.4762 (7.4747–72,171.0235)
1-Hydroxyphenanthrene	-0.3436	0.7454	-0.4609	.6449	0.7092 (0.1645–3.0571)	-3.0907	1.3474	-2.2938	**.0218**	0.0455 (0.0032–0.6378)
1-Hydroxypyrene	-0.7122	0.7367	-0.9667	.3337	0.4906 (0.1158–2.0788)	-4.0142	1.2892	-3.1136	**.0018**	0.0181 (0.0014–0.2260)

Bold values indicate *P* < .05.

CVD = cardiovascular disease.

**Table 3:** Association between 2-hydroxyfluorene in PAHs mixtures and the prevalence of CVDs and each specific CVD assessed by logistic model (adjusted for all covariates).

Variables	Single factor					Multiple factor				
	β	S.E	Z	*P*	OR (95%CI)	β	S.E	Z	*P*	OR (95%CI)
*Cardiovascular diseases*										
1-Hydroxynaphthalene	1.6727	0.447	3.7423	**.0002**	5.3268 (2.2182–12.7919)	-0.7602	0.7314	-1.0394	.2986	0.4676 (0.1115–1.9607)
2-Hydroxynaphthalene	0.3126	0.3526	0.8867	.3753	1.3670 (0.6850–2.7281)	0.6118	0.5931	1.0316	.3022	1.8438 (0.5766–5.8957)
3-Hydroxyfluorene	0.6116	0.3183	1.9211	.0547	1.8433 (0.9877–3.4400)	-0.6443	1.3978	-0.461	.6448	0.5250 (0.0339–8.1276)
2-Hydroxyfluorene	0.9481	0.3478	2.726	**.0064**	2.5808 (1.3053–5.1029)	4.7231	1.5991	2.9536	**.0031**	112.5201 (4.8986–2584.5584)
1-Hydroxyphenanthrene	0.1409	0.4533	0.3108	.756	1.1513 (0.4735–2.7991)	-3.6572	0.9411	-3.8859	**.0001**	0.0258 (0.0041–0.1632)
1-Hydroxypyrene	-0.7855	0.4499	-1.7458	.0809	0.4559 (0.1888–1.1012)	0.2374	0.8919	0.2662	.7901	1.2680 (0.2208–7.2828)
*Congestive heart failure*										
1-Hydroxynaphthalene	2.1365	0.7701	2.7741	**.0055**	8.4696 (1.8720–38.3188)	-0.7598	1.3032	-0.583	.5599	0.4678 (0.0364–6.0165)
2-Hydroxynaphthalene	1.3833	0.6452	2.1439	**.032**	3.9879 (1.1260–14.1240)	1.8919	1.0369	1.8246	.0681	6.6318 (0.8690–50.6086)
3-Hydroxyfluorene	1.0723	0.5671	1.891	.0586	2.9222 (0.9616–8.8798)	-0.421	2.4634	-0.1709	.8643	0.6564 (0.0053–82.0356)
2-Hydroxyfluorene	1.3972	0.6223	2.2452	**.0248**	4.0437 (1.1942–13.6922)	3.6897	2.8019	1.3169	.1879	40.0314 (0.1650–9712.9770)
1-Hydroxyphenanthrene	-0.0677	0.8262	-0.0819	.9347	0.9345 (0.1851–4.7187)	-4.3143	1.6735	-2.5779	**.0099**	0.0134 (0.0005–0.3555)
1-Hydroxypyrene	-0.3863	0.813	-0.4751	.6347	0.6796 (0.1381–3.3438)	-0.2236	1.5844	-0.1411	.8878	0.7996 (0.0358–17.8449)
*Myocardium infarction*										
1-Hydroxynaphthalene	0.5136	0.6771	0.7584	.4482	1.6712 (0.4433–6.3009)	-0.8219	1.0721	-0.7667	.4433	0.4396 (0.0538–3.5941)
2-Hydroxynaphthalene	-1.3234	0.5139	-2.575	**.01**	0.2662 (0.0972–0.7290)	-0.514	0.8403	-0.6117	.5408	0.5981 (0.1152–3.1049)
3-Hydroxyfluorene	-0.3379	0.4798	-0.7044	.4812	0.7132 (0.2785–1.8265)	0.239	2.0633	0.1158	.9078	1.2700 (0.0223–72.4548)
2-Hydroxyfluorene	0.0269	0.5161	0.0521	.9584	1.0273 (0.3735–2.8250)	4.6166	2.2955	2.0111	**.0443**	101.1460 (1.1247–9096.5361)
1-Hydroxyphenanthrene	-0.0781	0.6595	-0.1185	.9057	0.9248 (0.2539–3.3684)	-1.413	1.318	-1.072	.2837	0.2434 (0.0184–3.2231)
1-Hydroxypyrene	-2.8253	0.6812	-4.1476	**<.0001**	0.0593 (0.0156–0.2253)	-3.289	1.2361	-2.6608	**.0078**	0.0373 (0.0033–0.4205)
*Angina pectoris*										
1-Hydroxynaphthalene	1.4881	0.8133	1.8298	.0673	4.4289 (0.8995–21.8055)	-0.0287	1.2339	-0.0233	.9814	0.9717 (0.0866–10.9092)
2-Hydroxynaphthalene	-0.2547	0.6507	-0.3913	.6956	0.7752 (0.2165–2.7753)	-1.0371	1.055	-0.983	.3256	0.3545 (0.0448–2.8030)
3-Hydroxyfluorene	0.6122	0.5852	1.0461	.2955	1.8445 (0.5858–5.8080)	-0.9678	2.4719	-0.3915	.6954	0.3799 (0.0030–48.2830)
2-Hydroxyfluorene	0.9682	0.6382	1.517	.1293	2.6332 (0.7538–9.1990)	4.9468	2.7882	1.7742	.076	140.7203 (0.5957–33,240.5713)
1-Hydroxyphenanthrene	0.1577	0.8369	0.1884	.8505	1.1708 (0.2270–6.0378)	-3.7033	1.6054	-2.3068	**.0211**	0.0246 (0.0011–0.5731)
1-Hydroxypyrene	-0.3806	0.8251	-0.4613	.6446	0.6834 (0.1356–3.4439)	1.2847	1.5489	0.8295	.4068	3.6137 (0.1736–75.2275)
*Coronary heart disease*										
1-Hydroxynaphthalene	1.7568	0.6816	2.5774	**.01**	5.7939 (1.5233–22.0374)	-0.7024	1.1563	-0.6075	.5435	0.4954 (0.0514–4.7774)
2-Hydroxynaphthalene	-0.0375	0.5518	-0.068	.9458	0.9632 (0.3266–2.8405)	-1.0074	0.9245	-1.0897	.2758	0.3652 (0.0596–2.2356)
3-Hydroxyfluorene	1.1353	0.4877	2.3276	**.0199**	3.1121 (1.1964–8.0950)	-0.5633	2.1408	-0.2631	.7925	0.5693 (0.0086–37.8095)
2-Hydroxyfluorene	1.5531	0.5347	2.9048	**.0037**	4.7262 (1.6573–13.4781)	5.6173	2.3891	2.3512	**.0187**	275.1374 (2.5466–29,726.4229)
1-Hydroxyphenanthrene	0.6726	0.7064	0.9522	.341	1.9594 (0.4907–7.8237)	-2.7635	1.3945	-1.9816	**.0475**	0.0631 (0.0041–0.9702)
1-Hydroxypyrene	-0.3646	0.6993	-0.5213	.6021	0.6945 (0.1764–2.7348)	-0.445	1.324	-0.3361	.7368	0.6409 (0.0478–8.5859)
1-Hydroxynaphthalene	2.7073	0.6738	4.0182	**<.0001**	14.9890 (4.0018–56.1419)	0.3681	1.0309	0.3571	.721	1.4450 (0.1916–10.8997)
2-Hydroxynaphthalene	1.4655	0.5814	2.5205	**.0117**	4.3298 (1.3853–13.5326)	1.3333	0.9213	1.4472	.1478	3.7936 (0.6235–23.0804)
3-Hydroxyfluorene	1.1307	0.51	2.2173	**.0266**	3.0979 (1.1402–8.4167)	-0.5379	2.1444	-0.2508	.8019	0.5840 (0.0087–39.0596)
2-Hydroxyfluorene	1.4516	0.5601	2.5919	**.0095**	4.2701 (1.4247–12.7985)	5.1795	2.4798	2.0887	**.0367**	177.5926 (1.3759–22,922.0359)
1-Hydroxyphenanthrene	-0.3436	0.7454	-0.4609	.6449	0.7092 (0.1645–3.0571)	-5.2179	1.4859	-3.5116	**.0004**	0.0054 (0.0003–0.0997)
1-Hydroxypyrene	-0.7122	0.7367	-0.9667	.3337	0.4906 (0.1158–2.0788)	-0.5678	1.4005	-0.4054	.6852	0.5668 (0.0364–8.8210)

Bold values indicate *P* < .05.

CVD = cardiovascular disease.

### 3.3. Association between 2-hydroxyfluorene in PAHs and the prevalence of CVDs and each specific CVD assessed by BKMR model

**Figure 2.**
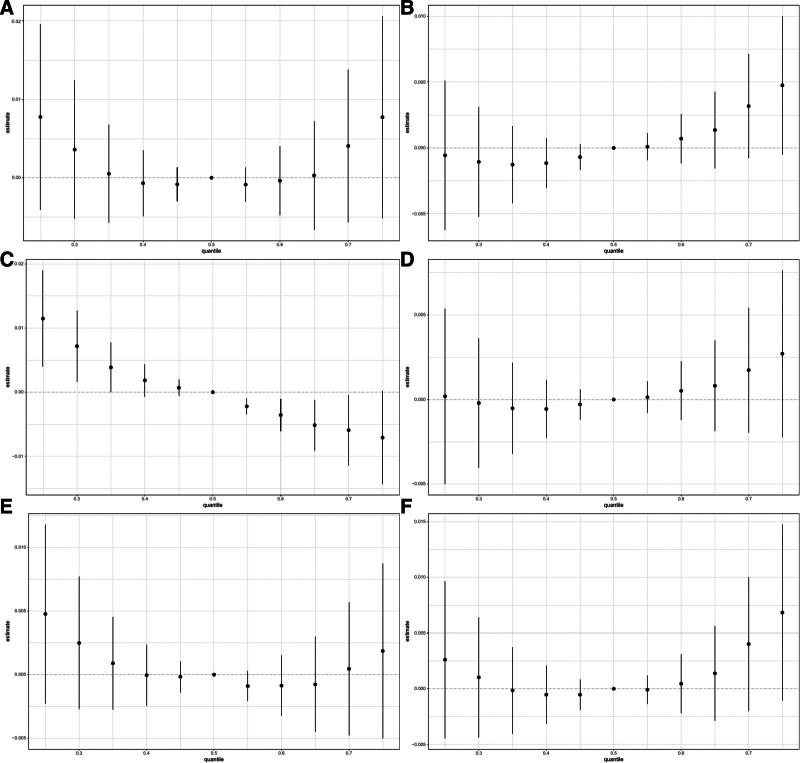
Overall effect of PAHs mixtures on CVD in BKMR model where all PAHs at specific percentiles were compared to their 50th percentile. (A) total CVD, (B) congestive heart failure, (C) myocardium infarction, (D) angina pectoris, (E) coronary heart disease, and (F) stroke. BKMR = Bayesian Kernel Machine Regression, CVD = cardiovascular disease, PAH = polycyclic aromatic hydrocarbon, URXP01 = 1-hydroxynaphthalene, URXP02 = 2-hydroxynaphtalene, URXP03 = 3-hydroxyfluorene, URXP04 = 2-hydroxyfluorene, URXP06 = 1-hydroxyphenanthrene, URXP10 = 1-hydroxypyrene.

**Figure 3.**
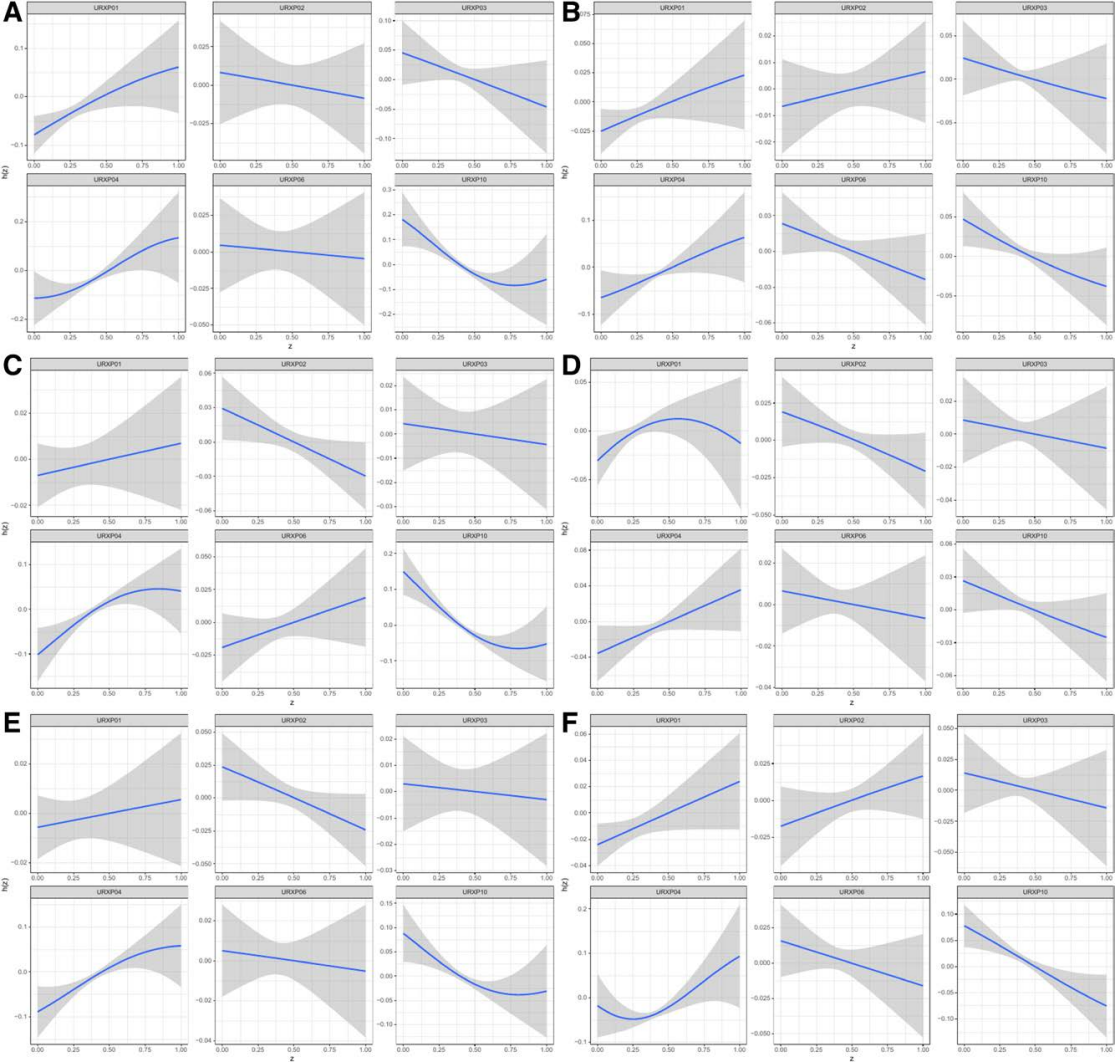
Univariate exposure–response function between each PAH and CVD when the other PAHs were fixed at 50th percentiles. (A) total CVD, (B) congestive heart failure, (C) myocardium infarction, (D) angina pectoris, (E) coronary heart disease, and (E) stroke. CVD = cardiovascular disease, PAH = polycyclic aromatic hydrocarbon, URXP01 = 1-hydroxynaphthalene, URXP02 = 2-hydroxynaphtalene, URXP03 = 3-hydroxyfluorene, URXP04 = 2-hydroxyfluorene, URXP06 = 1-hydroxyphenanthrene, URXP10 = 1-hydroxypyrene.

**Figure 4.**
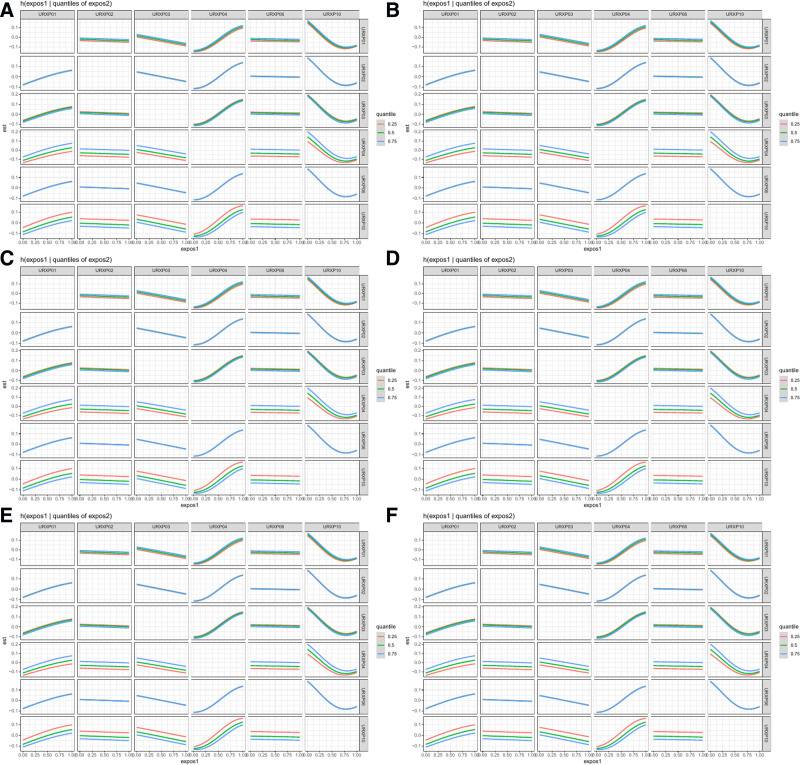
Single exposure-response functions for each PAH and CVD when a single PAH was at the 75th compared with the 50th percentile and the concentrations of all the other PAHs were fixed at either the 25th, 50th, 75th percentile in the BKMR model. (A) total CVD, (B) congestive heart failure, (C) myocardium infarction, (D) angina pectoris, (E) coronary heart disease, and (F) stroke. BKMR = Bayesian Kernel Machine Regression, CVD = cardiovascular disease, PAH = polycyclic aromatic hydrocarbon, URXP01 = 1-hydroxynaphthalene, URXP02 = 2-hydroxynaphtalene, URXP03 = 3-hydroxyfluorene, URXP04 = 2-hydroxyfluorene, URXP06 = 1-hydroxyphenanthrene, URXP10 = 1-hydroxypyrene.

**Figure 5.**
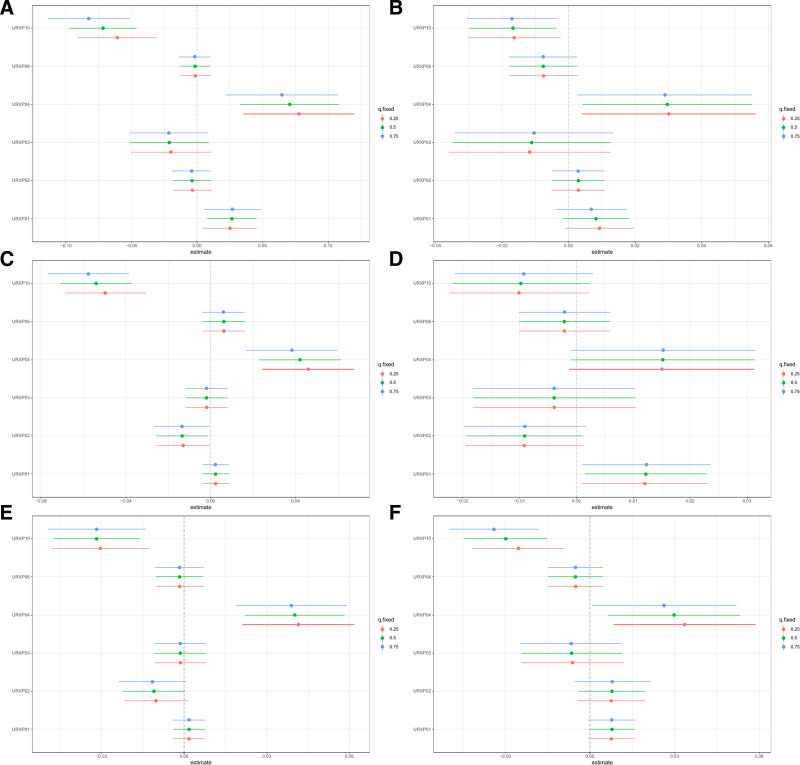
Bivariate exposure-response functions for each PAH and CVD when one PAH was fixed at 25th, 50th, 75th percentiles and other PAHs were fixed at the median in the BKMR model. (A) total CVD, (B) congestive heart failure, (C) myocardium infarction, (D) angina pectoris, (E) coronary heart disease and (F) stroke. BKMR = Bayesian Kernel Machine Regression, CVD = cardiovascular disease, PAH = polycyclic aromatic hydrocarbon, URXP01 = 1-hydroxynaphthalene, URXP02 = 2-hydroxynaphtalene, URXP03 = 3-hydroxyfluorene, URXP04 = 2-hydroxyfluorene, URXP06 = 1-hydroxyphenanthrene, URXP10 = 1-hydroxypyrene.

The BKMR model indicated a U-shaped effect on total CVD and each specific CVD, except for myocardial infarction which showed an inverted U-shaped effect (Fig. 2). Despite varied effects, 2-hydroxyfluorene consistently correlated positively with the prevalence of total and each specific CVD, excluding stroke (Fig. 3). No significant interaction effects were detected (Fig. 4). Configurations setting one phthalate at the 25th, 50th, and 75th percentiles while keeping others at their median displayed a constant positive association for 2-hydroxyfluorene with total and specific CVD prevalence (Fig. 5). 2-Hydroxyfluorene also demonstrated the highest probability of inclusion values among the PAH mix, strongly associating it with CVD prevalence (Table 4).

**Table 4:** PIPs of BKMR model.

	Total CVD	Congestive heart failure	Myocardium infarction	Angina pectoris	Coronary heart disease	Stroke
1-Hydroxynaphthalene	0.92	0.80	0.26	0.46	0.26	1.00
2-Hydroxynaphthalene	0.54	0.22	0.98	0.80	0.98	0.90
3-Hydroxyfluorene	0.14	0.46	0.34	0.60	0.34	0.40
2-Hydroxyfluorene	1.00	0.82	1.00	0.62	1.00	1.00
1-Hydroxyphenanthrene	0.30	0.76	0.86	0.16	0.70	0.58
1-Hydroxypyrene	1.00	0.48	1.00	0.66	1.00	1.00

CVD = cardiovascular disease, PIP = probability of inclusion.

### 3.4. Mediated effect of LAP index on the association between 2-hydroxyfluorene and prevalence of CVDs and each specific CVD

Mediation analysis assessed the LAP index’s role in mediating the association between 2-hydroxyfluorene and the prevalence of total CVD and each specific CVD. It confirmed that the LAP index mediated the relationship for total CVD, congestive heart failure, myocardial infarction, coronary heart disease, and stroke, with mediation proportions of 12%, 10%, 100%, 5%, and 9%, respectively (Table 5).

**Table 5:** Mediated effect of LAP index on the association between 2-hydroxyfluorene and prevalence of CVDs and each specific CVD.

Independent variable	Intermediary variable	Predictor variable	Direct effects β (95% CI)			Indirect effects β (95% CI)			Total effects β (95% CI)			Mediated proportion	*P*-value
			Estimate	CI lower	CI upper	Estimate	CI lower	CI upper	Estimate	CI lower	CI upper		
2-Hydroxyfluorene	LAP	Cardiovascular diseases	0.0117	0.0017	0.0217	0.0016	0.0005	0.0028	0.0133	0.0033	0.0234	0.12	**.0069**
2-Hydroxyfluorene	LAP	Congestive heart failure	0.0054	-0.0002	0.0110	0.0006	0.0001	0.0011	0.0060	0.0005	0.0116	0.10	**.0110**
2-Hydroxyfluorene	LAP	Myocardium infarction	-0.0004	-0.0064	0.0057	0.0005	0.0001	0.0010	0.0002	-0.0058	0.0061	1.00	**.0300**
2-Hydroxyfluorene	LAP	Angina pectoris	0.0036	-0.0017	0.0090	0.0004	0.0000	0.0008	0.0040	-0.0013	0.0094	0.09	.0576
2-Hydroxyfluorene	LAP	Coronary heart disease	0.0086	0.0023	0.0149	0.0005	0.0000	0.0010	0.0091	0.0028	0.0154	0.05	**.0361**
2-Hydroxyfluorene	LAP	Stroke	0.0071	0.0002	0.0139	0.0007	0.0001	0.0013	0.0078	0.0009	0.0146	0.09	**.0149**

Bold values indicate *P* < .05.

CVD = cardiovascular disease.

## 4. Discussion

This study identified a correlation between levels of 2-hydroxyfluorene and the prevalence of CVD, with the LAP index acting as a mediator.

Increasing evidence has highlighted the role of PAHs in CVD development. Studies using rodent models suggest that PAHs contribute to cancer and atherosclerosis progression through inflammatory pathways and enhanced plaque formation.^[24]^ These effects may also result from disruptions in autonomic nervous system regulation and arterial function, including narrowing of blood vessels.^[25]^ At the metabolic level, PAHs are converted into active carcinogens that interfere with cellular structures and molecular signaling.^[26]^ The toxicity of PAHs is often driven by altered intracellular signaling pathways.^[27]^ Furthermore, research consistently shows a link between PAH metabolites in urine and OS markers, such as 8-hydroxydeoxyguanosine (8-OHdG), 8-iso-prostaglandin F2a (8-iso-PGF2a), and malondialdehyde.^[28–30]^

PAHs are metabolized by Cytochrome P450 enzymes, generating reactive oxygen species that affect endothelial cell functionality, creating an environment prone to inflammation and coagulation. This OS can lead to cellular damage, increased permeability, and reduced vascular responsiveness, which may increase the risk of hypertension.^[31,32]^ Additionally, PAHs may activate the aryl hydrocarbon receptor, which has the potential to lower blood pressure through aryl hydrocarbon receptor-dependent mechanisms.^[33]^

The LAP, introduced by Professor Kahn in 2005, is derived from triglyceride and waist circumference (WC) measurements.^[14]^ It has proven to be a reliable and simple predictor of insulin resistance, MS, and CVD risk, outperforming traditional BMI measurements. While BMI is commonly used to assess overall body condition, it does not distinguish between fat and muscle mass and is not suitable for individuals with high muscle mass.^[15–17]^ In contrast, while WC is often used, it does not accurately reflect visceral fat, which is closely linked to metabolic disorders.^[34–37]^ For example, individuals with familial hyperceliac disease may show normal BMI and WC values but exhibit elevated LAP values, indicating a significant deviation from expected norms.^[38]^ By combining triglyceride and WC, LAP provides a more accurate measure of central obesity, a key risk factor for chronic diseases such as hypertension, hyperglycemia, hyperlipidemia, and CVD, all of which contribute to overall and CVD-specific mortality.^[39]^ As a result, LAP has garnered attention in numerous studies for its predictive capability in various diseases.

Our results suggest that PAH exposure might increase CVD prevalence through pathways involving the LAP index.

This study has limitations. Firstly, the cross-sectional design of NHANES prevents establishing causality or assessing long-term effects,^[40–42]^ underscoring the need for longitudinal studies to further investigate the relationship between phthalates and CVD. Secondly, the reliance on self-reported questionnaires for CVD diagnosis can introduce biases; future studies should incorporate multiple diagnostic methods and clinical consultations for greater precision. Thirdly, using only a single urinary sample may limit the depth of the analysis; multiple samples over time could offer more comprehensive insights. Lastly, although numerous confounding factors were accounted for, additional variables should be included in future research to improve the study’s robustness.

## 5. Conclusion

Our research revealed a notable link between exposure to 2-hydroxyfluorene and increased rates of CVDs. The LAP index played a mediating role in this relationship, highlighting the potential hazards. In the future, more prospective studies were needed to confirm the specific association.

## Author contributions

**Conceptualization:** Yuzhe Kong.

**Data curation:** Xinling Tian, Tian Xiong, Xiaoyi Zhu, Yuzhe Kong.

**Formal analysis:** Xinling Tian, Tian Xiong, Qingsong Mao, Xiaoyi Zhu, Yuzhe Kong.

**Investigation:** Xinling Tian, Tian Xiong, Xiaoyi Zhu, Yuzhe Kong.

**Methodology:** Yuzhe Kong.

**Project administration:** Yuzhe Kong.

**Resources:** Yuzhe Kong.

**Software:** Yuzhe Kong.

**Supervision:** Yuzhe Kong.

**Validation:** Yuzhe Kong.

**Visualization:** Xinling Tian, Xiaoyi Zhu, Yuzhe Kong.

**Writing – original draft:** Zhuofan Deng, Qingsong Mao, Yuzhe Kong.

**Writing – review & editing:** Yuzhe Kong.
